# Enhancing Personality Assessment in the Selection Context: A Study Protocol on Alternative Measures and an Extended Bandwidth of Criteria

**DOI:** 10.3389/fpsyg.2021.643690

**Published:** 2021-03-10

**Authors:** Valerie S. Schröder, Anna Luca Heimann, Pia V. Ingold, Martin Kleinmann

**Affiliations:** Department of Psychology, University of Zurich, Zurich, Switzerland

**Keywords:** personality, criterion-related validity, behavior description interview, situational judgment test, organizational citizenship behavior, counterproductive work behavior, adaptive performance, performance

## Abstract

Personality traits describe dispositions influencing individuals' behavior and performance at work. However, in the context of personnel selection, the use of personality measures has continuously been questioned. To date, research in selection settings has focused uniquely on predicting task performance, missing the opportunity to exploit the potential of personality traits to predict non-task performance. Further, personality is often measured with self-report inventories, which are susceptible to self-distortion. Addressing these gaps, the planned study seeks to design new personality measures to be used in the selection context to predict a wide range of performance criteria. Specifically, we will develop a situational judgment test and a behavior description interview, both assessing Big Five personality traits and Honesty-Humility to systematically compare these new measures with traditional self-report inventories regarding their criterion-related validity to predict four performance criteria: task performance, adaptive performance, organizational citizenship behavior, and counterproductive work behavior. Data will be collected in a simulated selection procedure. Based on power analyses, we aim for 200 employed study participants, who will allow us to contact their supervisors to gather criterion data. The results of this study will shed light on the suitability of different personality measures (i.e., situational judgment tests and behavior description interviews) to predict an expanded range of performance criteria.

## Introduction

In today's fast-moving world, the demands placed on employees are constantly changing, as is the definition of job performance (Organ, 1988; Borman and Motowidlo, 1993; Spector and Fox, 2005; Griffin et al., 2007; Koopmans et al., 2011). For selection procedures in organizations, the constant change of demands placed on employees may pose a challenge, especially when it comes to choosing appropriate predictor constructs to predict a wide range of job performance criteria. In this regard, assessing broad personality traits in selection seems promising given that personality traits are relatively stable in the working-age population (Cobb-Clark and Schurer, 2012; Elkins et al., 2017) and—outside of the scope of selection research—personality traits (such as the Big Five; Goldberg, 1992) have been found to relate to diverse performance criteria (e.g., Barrick and Mount, 1991; Hurtz and Donovan, 2000; Judge et al., 2013).

However, personality traits have often been questioned as valid predictors of performance in the selection context, as past research found “that the validity of personality measures as predictors of job performance is often disappointingly low” (Morgeson et al., 2007, p. 693). Looking at current practice, selection research on personality traits has neglected two important points that might explain these findings. First, selection research usually focuses on the prediction of task performance, but personality traits have been shown to be better at predicting non-task performance (Gonzalez-Mulé et al., 2014). Second, current practice in personnel selection often relies on self-report inventories as personality measures, which come with several limitations, especially in selection settings (Morgeson et al., 2007). Specifically, personality inventories are often not job-specific and they rely on self-reports, which can be distorted (Connelly and Ones, 2010; Shaffer and Postlethwaite, 2012; Lievens and Sackett, 2017).

There exist alternative measurement methods in personnel selection that do not have the same limitations as (personality) self-report inventories, but their suitability to measure personality has not yet been sufficiently studied (Christian et al., 2010). Two established measurement methods in personnel selection are situational judgment tests (SJTs; Christian et al., 2010) and behavior description interviews (BDIs; Janz, 1982; Huffcutt et al., 2001). In contrast to personality self-report inventories, SJTs and BDIs have the advantage that they are job-related, because they ask for applicants' behavior in specific situations on the job. Moreover, BDIs incorporate interviewers' evaluations of applicants. To date, few studies have developed personality SJTs or BDIs and even fewer have measured established personality traits such as the Big Five (Goldberg, 1992). The few studies that exist, however, suggest that SJTs and BDIs might be useful for measuring personality (Van Iddekinge et al., 2005; Oostrom et al., 2019; Heimann et al., 2020). Accordingly, more research on complementary measurements of personality is needed to foster this initial evidence and to systematically compare these new measures with each other.

The aim of this study is twofold: (1) expand the range of criteria predicted in selection contexts, shifting the focus to non-task performance, and (2) help to identify suitable approaches to assess personality in selection by systematically comparing different measurement methods that assess identical personality traits. To this end, we will develop SJTs and BDIs to measure the same personality traits (i.e., the Big Five personality traits, including Extraversion, Agreeableness, Conscientiousness, Emotional Stability, and Openness/Intellect and in addition Honesty-Humility; Goldberg, 1990; Ashton and Lee, 2009) and compare them with self-report inventories assessing the same traits regarding their prediction of task performance, adaptive performance, organizational citizenship behavior (OCB), and counterproductive work behavior (CWB; Koopmans et al., 2011). Simultaneously investigating several performance criteria will allow us to examine which outcomes are best predicted by personality constructs. Assessing the same traits with each measurement method will allow us to directly compare these methods and their suitability to measure each trait.

## Personality and Performance

Conceptually, personality is thought to drive individual job performance by influencing (a) what individuals consider to be effective behavior in work-related situations (knowledge), (b) to what extent they have learned to effectively engage in this behavior (skills), and (c) to what extent they routinely demonstrate this behavior at work (habits; Motowidlo et al., 1997). For example, individuals high in Agreeableness might strive to cooperate with others in everyday life. Thus, they are more likely to know which behaviors are effective at enabling cooperation (e.g., actively listening to others and asking questions to better understand them) and how to effectively display these behaviors (Motowidlo et al., 1997; Hung, 2020). When it comes to working in a team, agreeable individuals are thus more likely to cooperate successfully with others, based on their knowledge, skills and habits (Tasa et al., 2011).

Although personality predicts job performance, it does not seem to be the best predictor of the aspect personnel selection usually focuses on. The most common aspect of job performance is task performance, which is defined as the competency to fulfill central job tasks (Campbell, 1990). Personality traits can predict task performance, with Conscientiousness and Emotional Stability being the strongest predictors among the Big Five traits (Barrick et al., 2001; He et al., 2019). Yet, the fulfillment of job tasks seems to depend largely on mental processes, as recent meta-analytic evidence found that cognitive ability predicts task performance better than personality (Gonzalez-Mulé et al., 2014).

Personnel selection could particularly benefit from personality traits as predictors when expanding the range of criteria to include non-task performance. Non-task performance consists of behaviors that do not directly contribute to the main goal of the organization (Rotundo and Sackett, 2002) and can be specified into three aspects: adaptive performance, OCB, and CWB (Koopmans et al., 2011). In contrast to task performance, non-task performance might depend largely on motivation or personality and less on general mental ability. In line with this, numerous personality traits have been linked to the three forms of non-task performance (Barrick and Mount, 1991; Dalal, 2005; Judge et al., 2013; Huang et al., 2014; He et al., 2019; Lee et al., 2019; Pletzer et al., 2019). Yet, only a few of the studies linking personality to non-task performance have been conducted in personnel selection research [i.e., empirical studies that either simulate a selection procedure or use actual applicants as a sample; see for example Dilchert et al. (2007), Lievens et al. (2003), Swider et al. (2016), and Van Iddekinge et al. (2005)]. Yet, the studies conducted so far suggest that different personality traits predict different types of non-task performance.

*Adaptive performance* can be described as “behaviors individuals enact in the response to or anticipation of changes relevant to job-related tasks” (Jundt et al., 2015, p. 55). In contrast to task-based performance, adaptive performance implies that employees adapt to changes beyond the regular fulfillment of work tasks (Lang and Bliese, 2009; Jundt et al., 2015). In accordance with this, adaptive performance can describe reactive behaviors such as coping with changes in core tasks (Griffin et al., 2007) and relearning how to perform changed tasks (Lang and Bliese, 2009). Going beyond reactive behavior, some researchers also highlight the relevance of proactive behaviors for adaptive performance such as producing new ideas or taking initiative (Griffin et al., 2007).^1^ Research outside of personnel selection has shown that reactive adaptive performance is related to Emotional Stability (e.g., being unenvious, relaxed, unexcitable; Huang et al., 2014), whereas proactive adaptive performance is thought to relate to Openness/Intellect (e.g., being creative, imaginative, innovative) as well as Extraversion (e.g., being talkative, assertive, bold; Marinova et al., 2015). Empirical findings for Conscientiousness (e.g., being organized, efficient, goal-oriented) are mixed (Jundt et al., 2015). Yet, conceptually, conscientious individuals strive for success and are thus likely to show proactive behavior (Roberts et al., 2005). Even though the rapid changes in the work environment today require individuals to show adaptive performance (Griffin and Hesketh, 2003) personnel selection research has rarely considered this form of non-task performance as a criterion (Lievens et al., 2003).

*OCB* describes individual behavior outside the formally prescribed work goals (Borman and Motowidlo, 1993) and has been shown to contribute to an organization's performance (Podsakoff et al., 2009). Research distinguishes between OCB directed at other individuals (e.g., helping newcomers; OCB-I) and OCB directed at the organization (e.g., taking extra tasks or working overtime; OCB-O). Research outside of personnel selection has shown that personality is particularly suited to predict this type of non-task performance. Whereas, some studies have found that OCB-I and OCB-O are predicted equally well by Conscientiousness, and Agreeableness (being kind, sympathetic, warm; Chiaburu et al., 2011; Judge et al., 2013), other results suggest that OCB-I is best predicted by Agreeableness and OCB-O is best predicted by Conscientiousness (Ilies et al., 2009). Despite the relevance of OCB for organizations, there exist only a few studies on its relationship with personality in selection research (Anglim et al., 2018; Heimann et al., 2020).

*CWB* is defined as actions that harm the legitimate interests of an organization (Bennett and Robinson, 2000) and either damage other members of the organization (CWB directed at other individuals such as bullying co-workers; CWB-I) or the organization itself (CWB directed at the organization such as theft or absenteeism; CWB-O). Research outside of personnel selection has found some evidence that, overall, CWB is best predicted by Conscientiousness, Agreeableness (He et al., 2019), Honesty-Humility (e.g., being sincere, fair, and modest; de Vries and van Gelder, 2015; Lee et al., 2019), and Emotional Stability (Berry et al., 2007). Going beyond the traditional Big Five personality traits, Honesty-Humility has been shown to explain a significant proportion of variance in CWB over and above the other personality traits (Pletzer et al., 2019). Despite its harm to organizational success (Berry et al., 2007), CWB has rarely been considered as a criterion in selection research (Dilchert et al., 2007; Anglim et al., 2018).

### Assessing Personality in the Selection Context

Personality is typically assessed via self-report inventories, which face three major limitations in the selection context: (1) a lack of contextualization, (2) relying on applicants as the only source of information, and (3) a close-ended response format (Connelly and Ones, 2010; Oh et al., 2011; Shaffer and Postlethwaite, 2012; Lievens and Sackett, 2017; Lievens et al., 2019). *Contextualization* describes the degree to which a measurement method refers to a specific situation or context, such as the work context. The problem of generic (i.e., non-contextualized) personality inventories is that people do not necessarily behave consistently across different contexts (Mischel and Shoda, 1995). The same person might show different behavior at work compared to in their free time. In generic personality inventories, the same applicant might apply a different frame-of-reference when replying to different items, causing within-person inconsistency. Within-person inconsistency has been shown to affect the reliability and validity of personality inventories (Lievens et al., 2008). Further, different applicants might think of very different situations when replying to the same generic item, thereby increasing the between-person variability. Between-person variability has been shown to affect the validity of personality inventories (Lievens et al., 2008). In addition, when applicants complete a personality measure without referring to the context of work, there will be a mismatch with the criteria that we want to predict in selection contexts (i.e., performance and behavior *at work*). A simple way to address this problem is to contextualize inventories by adding the term “at work” to every generic item. Although the change is minor, adding this frame-of-reference increases the validity of personality inventories (Lievens et al., 2008; Shaffer and Postlethwaite, 2012).

The *source of information* refers to the person who responds to the personality inventory (Lievens and Sackett, 2017). Personality inventories rely only on one information source, namely the self-report of applicants. The use of one-sided information can lead to inaccurate assessments because the target group of applicants has a specific interest to present themselves most favorably and to potentially distort their answers (Ellingson and McFarland, 2011). Research has shown that assessing personality in applicant samples leads to different factor structures compared to non-applicant samples (Schmit and Ryan, 1993). Furthermore, one's own self-perception can differ from the perception of others (McAbee and Connelly, 2016). Thus, answers can be distorted not only through intentional self-distortion but also through self-evaluations, which might not completely represent a person. It is therefore not surprising that personality traits are better predictors when they are assessed via other-reports compared to self-reports (Oh et al., 2011).

The *response format* describes whether a measurement method provides predefined response options (Lievens and Sackett, 2017). Personality inventories use a close-ended response format. Close-ended response formats do not allow applicants to generate their answer freely. Thus, they provide a smaller information base to assess the applicant's personality compared to open-ended response formats, in which applicants can generate detailed answers and get the opportunity to share additional information about themselves. Furthermore, close-ended response formats may facilitate response distortion, because a limited number of presented response options makes them more transparent than open-ended formats. In a closed-ended response format, applicants might identify or guess the “right” or most desired response option and can thus more easily direct their response in the intended direction.

SJTs and BDIs could be used as alternative or complementary measurement methods to help overcome the limitations of personality measurement in personnel selection. SJTs and BDIs are established instruments in personnel selection and have been shown to predict job performance (Christian et al., 2010; Culbertson et al., 2017). Both measurement methods provide a precise frame-of-reference and thus have a high contextualization.

In SJTs, short work-related situations are presented to applicants along with several response options, describing possible behaviors in this situation. Applicants are asked to choose the response option that most likely describes their own behavior in this situation (Mussel et al., 2018). In comparison to contextualized self-report personality inventories, SJTs are more contextualized because they present a clear frame-of-reference for behavior by describing a specific work-related situation. Yet, like personality inventories, they rely on only self-reports and have a close-ended response format.

In BDIs, applicants receive descriptions of situations that employees have typically experienced within the context of work (Janz, 1982). Interviewers present the description and ask applicants to describe a corresponding or similar situation in their past working experience, and to report their personal feelings and behavior in this situation. Responses are rated on behaviorally anchored rating scales (Klehe and Latham, 2006). BDIs are a popular method in personnel selection and can predict performance across different domains (Culbertson et al., 2017). BDIs have three advantages over SJTs. First, interviewers serve as an additional information source, because they can specify, interpret, and evaluate the information provided by the applicant. Second, BDIs use an open-ended response format, which allows applicants to share more information of themselves and thereby provide a richer information base (Van Iddekinge et al., 2005; Raymark and Van Iddekinge, 2013). As interviewees' answers are rated directly after the interview on behaviorally anchored rating scales, this results in a quantitative data format. Third, the cognitive demand of BDIs should make them the least prone to self-distortion. Both BDIs and SJTs place higher cognitive demands on applicants than personality inventories and should thus reduce response distortion (Sweller, 1988; Sackett et al., 2017) because they require the applicant to process more information. In BDIs, applicants simultaneously describe situations and interact with interviewers, causing high cognitive demand. To distort their answers, applicants would need to fabricate past situations in a short time-frame while monitoring their own behavior to appear truthful and also preparing to answer follow-up questions (Van Iddekinge et al., 2005). Table 1 presents an overview of different features of self-report inventories, SJTs, and BDIs regarding contextualization, information source (self- vs. other-rating), and response format.

**Table 1:** Characteristics of personality measures adapted from Heimann and Ingold (2017) and Lievens and Sackett (2017).

	**Generic personality inventory**	**Contextualized personality inventory**	**Situational judgment test**	**Behavior description interview**
Contextualization	Low levels of contextualization	Low to medium levels of contextualization	Medium levels of contextualization (brief descriptions of task, characters, etc.)	High levels of contextualization (reference to tasks and characters in previously experienced situations)
Information source	Applicant	Applicant	Applicant	Applicant and trained interviewers
Response format	Close-ended	Close-ended	Close-ended	Open-ended

### Aims and Hypotheses

**Figure 1:**
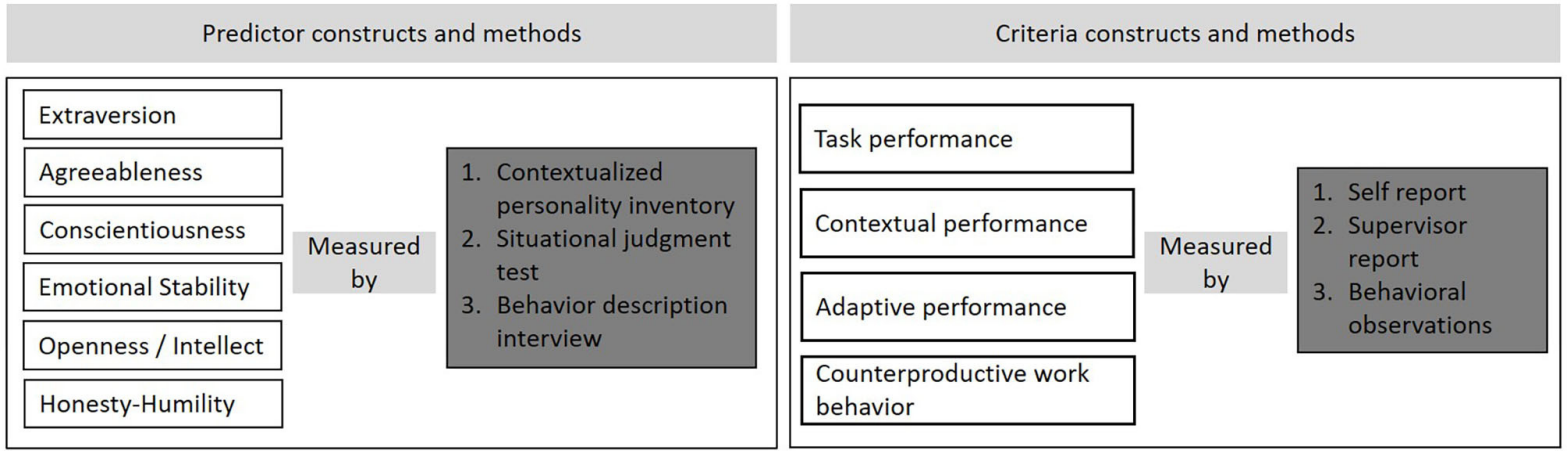
Overview of constructs and measures.

The overall objective of this study is twofold: (1) to widen and shift the focus of selection research from solely predicting task performance to predicting other relevant performance criteria; and (2) to identify suitable measurement methods assessing personality to predict these criteria. Therefore, we will develop an SJT and BDI to measure the Big Five personality traits and Honesty-Humility. As depicted in Figure 1, we will use the Big Five traits and Honesty-Humility measured by a contextualized personality inventory, an SJT, and a BDI to predict different performance criteria. We assume that personality traits will predict both task- and non-task performance criteria (task performance, adaptive performance, OCB, CWB) within a personnel selection setting. Specifically, we expect the same pattern of relationships between specific sets of personality traits with specific performance criteria as they have been found outside of personnel selection research (Barrick and Mount, 1991; Dalal, 2005; Judge et al., 2013; Huang et al., 2014; He et al., 2019; Lee et al., 2019; Pletzer et al., 2019). Regarding the comparison of personality measures, we predict that the criterion-related validity of personality measures will depend on (1) the contextualization of methods, such that more contextualization should lead to higher validity, (2) the source of information, such that other ratings (i.e., interviewer ratings) should be superior to self-reports, and (3) the response format, such that open-ended formats should be superior to close-ended formats. As a result, both the SJT and BDI should explain incremental validity in performance criteria over the contextualized personality inventory. BDIs should be superior to both the personality inventory and SJT.

## Methods and Analyses

### Participants

Participants will be employed individuals who are willing to participate in a simulated selection procedure to prepare and practice for their future job applications. We will recruit individuals who plan to apply for a new job and will contact them through universities and career services. Participants must be employed to participate, and they must name their supervisor so that we can collect supervisor performance ratings. Within the simulated selection procedure, participants can gain experience with established selection instruments and they will receive extensive feedback on their performance. A power analysis was conducted in G^*^Power (Faul et al., 2007) for hierarchical regression analyses with the conventional alpha level of α = 0.05 and power of 80. Based on previous results (Chiaburu et al., 2011; Heimann et al., 2020) we assume a mean correlation of 0.13 between personality predictors (measured with self-report inventories) and performance criteria and predict that measures of personality by alternative methods can explain between 4 and 5% of additional variance compared to traditional personality inventories. Further, we expect a participant dropout of 10%, based on experiences in previous studies. Accounting for dropout, the power analysis resulted in a total sample size of *N* = 200.

### Design

Data will be collected in a simulated selection procedure, allowing us to administer personality measures under controlled conditions and collect various performance data. Similar study designs have been successfully used in previous selection research (Van Iddekinge et al., 2005; Klehe et al., 2008; Kleinmann and Klehe, 2011; Ingold et al., 2016; Swider et al., 2016). The simulated one-day selection procedure will consist of different personality measures to assess personality predictors (i.e., a contextualized personality inventory, an SJT, a BDI), behavioral observations rated in work simulations and standardized situations during the day to assess performance criteria, and other measures. All participants will complete all measures. Measures will be presented in randomized order to control for order effects.

A panel of interviewers will evaluate participants' personality (i.e., Big Five traits and Honesty-Humility) in the BDI and an independent panel of assessors will evaluate performance dimensions (i.e., task performance, OCB, adaptive performance, and CWB) in proxy criteria (work simulations; e.g., group discussion, presentation exercise). Interviewers will only rate predictors (i.e., personality) and assessors will only rate criteria (i.e., job performance) to avoid rater-based common method variance between predictor and criteria. Interviewers and assessors will be industrial-organizational psychology graduate students who will receive rater training prior to participating in this study.

The simulated selection procedure will be designed as realistically as possible so that participants' behavior is as close as possible to their behavior in a real selection process. For example, participants will be asked to dress as they would for a job interview. To further motivate participants to perform well, the best participant of the day will receive a cash prize (CHF 100). Participants will fill out a manipulation check at the end of the simulated selection procedure. Similar to previous studies using this type of design, the manipulation check will contain questions asking how realistic participants perceived the selection procedure to be and whether they felt and acted like they would in a real selection procedure (Klehe et al., 2008; Jansen et al., 2013; Heimann et al., 2020). Participants will give their informed consent prior to participating in the simulated selection procedure. Their participation will be voluntary, and they will be allowed to quit at any time during the procedure.

### Measures

#### Personality

We will measure the broad Big Five personality traits (including Extraversion, Agreeableness, Conscientiousness, Emotional Stability, and Openness/Intellect) and Honesty-Humility as predictors in this study. The broad personality predictors will be assessed with three different measures: a contextualized self-report inventory, an SJT, and a BDI. In addition, given that former research suggests that narrow facets are useful for predicting specific behavior (Paunonen and Ashton, 2001), we will measure selected facets relevant for our criteria in the personality inventory (e.g., achievement striving, ingenuity).

For the contextualized personality inventory, we will use the 50-item IPIP representation of the Goldberg (1992) makers for the Big Five factor structure and the subscale “Honesty-Humility” from the HEXACO scale (Ashton and Lee, 2009) with all items adapted to the context of work [similar to Lievens et al. (2008) and Heimann et al. (2020)]. Items will be contextualized by adding the term “at work” at the beginning of each item (e.g., “At work, I am always prepared”). Internal consistencies for the original scales ranged between α = 0.76 (for Openness/Intellect) and α = 0.89 (for Emotional Stability) for the Big Five scale (Lievens et al., 2008) and between α = 0.74 and α = 0.79 for the Honesty-Humility Subscale (Ashton and Lee, 2009).

The SJT and BDI will be newly developed for this study. To allow for valid comparisons of personality measures, the SJT and BDI will be designed in *parallel* and they will be based and closely aligned with established personality self-report items. Thus, the SJT and BDI will contain similar, but not identical situations. Given that theory assumes that personality is only expressed if a situation contains certain situational cues that activate a trait (trait activation; Tett and Guterman, 2000), we will design situations to be equivalent in terms of the trait-activating cues.

The development of the SJT and BDI will proceed in four steps in line with previous studies that developed situation-based personality measures (Van Iddekinge et al., 2005; Mussel et al., 2018; Heimann et al., 2020). First, we will select items from the 100 item IPIP Big Five scale (Goldberg et al., 2006) and the Honesty-Humility subscale of the HEXACO model (Ashton and Lee, 2009) from different facets of each personality dimension to serve as the basis for SJT items and BDI questions. In case of the Big Five traits, we will ensure that the selected items cover both aspects of the model by DeYoung et al. (2007). The model indicates that each Big Five trait encompasses two distinct aspects, based on factor analytical results. For example, the personality dimension Conscientiousness encompasses the aspects Industriousness and Orderliness. By covering both aspects, we will ensure that the corresponding personality dimensions will be comprehensively measured. We will select items that (a) could be related to the criterion on the basis of conceptual and/or empirical arguments, (b) could be adapted to the working context, and (c) express an observable behavior.

Second, for each selected item, the first author of this study will generate situations that typically occur in working life and in which the respective traits would influence behavior; that is, situations in which a person who scores high on the item would behave differently compared to someone who scores low. Given that research shows that situations can be clustered into different types of situations based on the perceptions they elicit (e.g., Sherman et al., 2013; Rauthmann et al., 2014; Funder, 2016), and that these clusters are tied to certain traits (Rauthmann et al., 2014), we will systematically design different situations in order to ensure fit between the situation described and the trait we aim to activate (trait activation; Tett and Guterman, 2000). To reduce transparency and socially desirable responding, every situation will be designed to contain a dilemma, meaning that more than one response to the given situation would be socially desirable. For example, participants will have to think of a situation in which they are under time pressure at work and a co-worker asks for help with a different task. Thus, both concentrating on their own tasks in order to meet the deadline *and* helping the co-worker would be socially acceptable behaviors in this situation. To make the situation more specific, we included different examples in each SJT item and BDI question. Each situation is constructed to measure a single trait. For each item, the first author will generate one hypothetical situation (for the SJT) and one past-behavior/typical situation (for the BDI).

Third, for each SJT item the first author of this study will further generate five response options. Response options will represent behavioral alternatives in this situation. Behavioral alternatives will express five different gradations of the item. The dilemma presented in the situation description will be mentioned in each response option. For example, in case of the aforementioned situation, a response option corresponding to a high expression of Agreeableness could be “I will help my co-worker, even if it means that I cannot meet the deadline for my own tasks.” For each BDI item, the first author will develop behaviorally anchored rating scales expressing high, medium, and low expressions of the respective trait.

Fourth, the co-authors of this study will thoroughly review SJT items and BDI questions and the response options several times, with regard to (a) the fit between the described situation and the trait (Rauthmann et al., 2014); (b) their trait activation, that is, the strength of the cues that are assumed to activate the relevant behavior in the situation (Tett and Guterman, 2000); (c) the strength of the dilemma described in the situation, that is, whether the behavioral alternatives are equally socially desirable [see also Latham et al. (1980)]; (d) similar phrasing of items across measures. The co-authors are researchers in the field of I/O psychology with a focus on personnel selection or interview research. Based on these reviews, the first author will carefully revise the items several times. If necessary, situations will be newly developed and again reviewed and revised. We aim to design SJT items and BDI questions to be as parallel as possible by ensuring that all situations meet the aforementioned criteria (i.e., items and questions should describe a dilemma situation, provide specific examples, and not be too transparent). At the same time, we aim to keep SJT items and BDI questions as short as possible. As a pretest, a sample of at least four students will complete all SJT items and BDI questions to check the extent that they are comprehensible and how much time will be required to complete them. The first author of this study will then check whether the provided answers show variability in the respective traits and whether answers for BDI items correspond with the intended rating scales. The first author will then revise the items again based on the evaluation and the feedback provided by the test sample.

Samples for the SJT items and BDI questions are shown in Table 2. Past studies on personality-based SJTs have reported internal consistencies between α = 0.55 and α = 0.75 (Mussel et al., 2018), and between α = 0.22 and α = 0.66 (Oostrom et al., 2019). Past studies on personality-based BDIs reported ICCs (interrater reliability) of 0.78 (Heimann et al., 2020) and 0.74 (Van Iddekinge et al., 2005).

**Table 2:** Sample items of situational judgment test and behavior description interview based on the conscientiousness item “I am always prepared.”

**Method**	**Sample Item**
Situational judgment test	Your organization has developed a new product. You are given the task of making the product known to various target groups. To do this, you have to deliver the same presentation to different professional and personal groups, each with different interests in relation to the product (e.g., representatives, advertising managers, internal, and external customers). At the same time, you also have the task of preparing reports on the product, which is very time-consuming. How would you personally experience this situation and how would you behave in this situation regarding the preparation for the presentations? **A**. Before each appointment, I take the time to revise the presentation for the respective target group and include references to the respective needs of the target group. Whenever possible, I try not to neglect the other tasks. **B**. Although I also have to concentrate on the other tasks, I re-prepare the presentation before each appointment and adapt it slightly to the interests of the target group. **C**. I take a brief look at the presentation before each appointment to re-prepare for it. On the way to the respective presentation appointment, I consider how I could link the presentation to the target group. However, I do not make any major changes in the presentation, as I have to concentrate on the other tasks. **D**. As soon as I have the presentation somewhat in mind and know its content, I no longer look at it before the appointments because I don't want to neglect the other tasks. **E**. Since I don't want to get stressed by the deadlines of my other tasks, I don't prepare myself for the individual presentations. Since I have to give them several times anyway, the preparation comes naturally.
	**Sample Question**
Behavior description interview	*Situation description:* Think of a situation where you had a similar conversation with different people or where you had to explain the same thing to different people. This could be a job interview, the induction of a new employee, or the repeated explanation of a certain work process. It was not absolutely necessary to re-prepare for every situation and you had other tasks to do. Please tell us briefly in one or two sentences what the situation was. Then report how you personally experienced the situation and how you behaved in this situation regarding the preparation for the conversations. *Behavioral anchors (not presented to the interviewee; rating on a 1–5 scale)*: **5**—re-prepares for each conversation (e.g., takes another look at materials, adapts the conversation to the person), inquires about each individual appointment (e.g., gets information about the person), finds it important to always be prepared, feels more secure 3—invests a certain amount of re-preparation time before each conversation (e.g., takes another look at materials), invests only as much as necessary, does not want to invest too much time (e.g., does not adjust anything), does not want to be unprepared 1—does not invest any further preparation effort (e.g., does not take another look at it), does not want to invest unnecessary time (i.e., beyond the duration of the appointment), feels safe even without additional preparation

*The answer reflecting the highest expression of the trait is marked bold*.

#### Performance

All performance criteria (i.e., task performance, adaptive performance, OCB, and CWB) will be assessed with three different measurement approaches: self-reports, supervisor ratings, and proxy criteria. Self-reports and supervisor ratings will be assessed with established scales for all performance criteria. For task performance, we will use items by Bott et al. (2003) and Williams and Anderson (1991). This composite scale has been used in previous studies and showed a reliability of α = 0.92 (Jansen et al., 2013). For adaptive performance, we will use the individual task adaptivity and individual task proactivity scales from Griffin et al. (2007). Reliability of the scales range from α = 0.88 to α = 0.89 for adaptivity and from α = 0.90 to α = 0.92 for proactivity (Griffin et al., 2007). For OCB, we will use the OCB-I and OCB-O scales from Lee and Allen (2002). Reliabilities of the scales were between α = 0.83 and α = 0.88. For CWB, we will use the workplace deviance scale from Bennett and Robinson (2000) with reliabilities ranging from α = 0.78 to α = 0.81. Example items for all measures can be found in Table 3. We will use the same scales with small adaptations in items for both self-reports and supervisor ratings of performance criteria.

**Table 3:** Main measures.

**Construct**	**Type of measure**	**Sample item**
Big Five personality traits (Extraversion, Agreeableness, Conscientiousness, Emotional Stability, Openness/Intellect) and Honesty Humility	Contextualized self-reported inventory (adapted items IPIP Big Five and Honesty-Humility; Goldberg et al., 2006; Ashton and Lee, 2009)	“At work, I am always prepared”
	Situational Judgment Test (self-developed)	see Table 2
	Behavior Description Interview (self-developed)	see Table 2
Task Performance	Self-report [(composite scale with items by Bott et al. (2003) and Williams and Anderson (1991)]	“I achieve the objectives of the job”
	Supervisor report [composite scale with items by Bott et al. (2003) and Williams and Anderson (1991)]	“He/She achieves the objectives of the job”
	Proxy criteria: Performance in assessment center exercises: presentation exercise, role play, group discussion, business simulation	
Adaptive Performance- Reactive	Self-report [individual task adaptivity scale from Griffin et al. (2007)]	“I adapt well to changes in core tasks”
	Supervisor report [individual task adaptivity scale from Griffin et al. (2007)]	“He/She adapts well to changes in core tasks”
	Proxy criteria: showing adaptability in unexpected situations during assessment center exercises: presentation exercise, role play, business simulation	
Adaptive Performance- Proactive	Self-report [individual task proactivity scale from Griffin et al. (2007)]	“I initiate better ways of doing my core tasks”
	Supervisor report [individual task proactivity scale from Griffin et al. (2007)]	“He/She initiates better ways of doing his/her core tasks”
	Proxy criteria: showing initiative and suggesting new ideas in assessment center exercises: group discussion, business simulation; making improvement suggestions for the project	
OCB-I	Self-report [OCB scale form Lee and Allen (2002)]	“I help others who have been absent”
	Supervisor report [OCB scale form Lee and Allen (2002)]	“He/She helps others who have been absent”
	Proxy criteria: helping other individuals in various interactions during the selection procedure	
OCB-O	Self-report [OCB scale form Lee and Allen (2002)]	“I express loyalty toward the organization”
	Supervisor report [OCB scale form Lee and Allen (2002)]	“He/She expresses loyalty toward the organization”
	Proxy criteria: supporting the project by recommending it to others, helping with administrative tasks during the selection procedure	
CWB-I	Self-report workplace deviance scale Bennett and Robinson, 2000	“I make fun of someone at work”
	Supervisor report	“He/She makes fun of someone at work”
	Proxy criteria: acting unkindly toward other individuals in interactive assessment center exercises: role play, group discussion, business simulation	
CWB-O	Self-report workplace deviance scale Bennett and Robinson, 2000	“I come in late to work without permission”
	Supervisor report	“He/She comes in late to work without permission”
	Proxy criteria: harming the project's success with uncooperative behavior, e.g., lying about test results, stealing assessment center exercises, breaking rules	

*OCB-I, Organizational Citizenship Behavior Interpersonal; OCB-O, Organizational Citizenship Behavior Organizational; CWB-I, Counterproductive Work Behavior Interpersonal; CWB-O, Counterproductive Work Behavior Organizational*.

Proxy criteria will be behavioral observations rated in standardized situations during the selection procedure. More precisely, we will use (a) assessment center exercises, (b) standardized staged situations and, (c) compliance in the simulated selection procedure to assess participants' performance. For example, we will assess the performance of participants in a presentation exercise (i.e., whether the presentation is well-structured, whether it includes all relevant information) as a proxy criterion for task performance. As an example of a staged situation, interviewers will pick up each participant in a room for their interview, while carrying several items of material (e.g., folders). On the way to the interview room, interviewers will have difficulty opening the doors to the stairway due to the material they carry. Interviewers will observe whether participants help them to open the door as a proxy criterion for OCB. Behavior will be rated using behaviorally anchored rating scales. A more detailed description of proxy criteria for each performance dimension and an overview of all measures is presented in Table 3. We will use proxy criteria in addition to self-reports and supervisor ratings of all performance criteria to add a behavioral observation and to ensure that one source of performance ratings is assessed in a standardized setting. Such proxy criteria have already been successfully employed in previous studies in selection research (e.g., Kleinmann and Klehe, 2011; Klehe et al., 2014).

### Planned Analyses

Statistical analyses will be carried out using R. Data will be screened separately for each participant in order to identify spurious data. We will report all data exclusions (if any). We will first check whether applicants perceived the simulation setting as realistic. We will check plausibility of data with descriptive analysis using the *psych*-package for the R environment (Revelle and Revelle, 2015). We will also check if variables are normally distributed (especially for data on proxy criteria) and transform non-normally distributed data. All measures will be designed as interval scales, and we will additionally check whether they can be analyzed accordingly, depending on the actual distribution of the data on the scales. Otherwise, we will adjust the analysis accordingly (i.e., evaluate them with methods for ordinal data).

To investigate the extent to which the SJT items and the BDI questions accurately measure personality traits, we will first examine the internal data structure (i.e., construct-related validity) of the newly developed SJT and BDI using multitrait-multimethod analyses within and across methods (similar to Van Iddekinge et al., 2005). First, to conduct correlative analyses of the data structure, we will use the *psy*-package (Fallissard, 1996) and *multicon*-package (Sherman, 2015). Regarding analyses within methods (i.e., examining the internal data structure of the SJT and BDI separately), we will investigate whether SJT items or BDI questions measuring the same traits show stronger intercorrelations than SJT items or interview questions measuring different traits. Regarding analyses across methods (i.e., examining the data structure across the personality inventory, SJT, and BDI), we will investigate whether the same traits measured with different methods correlate to test for convergent validity (average monotrait-heteromethod correlation). Further, we will calculate the correlation of different traits assessed with the same method (average heterotrait-monomethod correlation) to test for divergent validity. Thereby, we will verify whether the different traits can be distinguished when measured with the same method (personality inventory, SJT or BDI).

Second, to further examine the latent data structure within and across methods with confirmatory factor analyses (CFAs), we will use the *lavaan* package (Rosseel, 2012). Regarding analyses within methods, we will conduct separate CFAs for each method (personality inventory, SJT and BDI). Regarding analyses across methods, we will conduct multitrait-multimethod CFAs on data from all three methods. The personality traits (Big Five traits plus Honesty-Humility) will be specified as latent trait factors and the three methods (personality inventory, SJT, and BDI) will be specified as latent method factors. Thereby, we will examine to what extent the different methods (personality inventory, SJT, and BDI) measure the same constructs.

Second, to further examine the latent data structure within and across methods with confirmatory factor analyses (CFAs), we will use the *lavaan* package (Rosseel, 2012). Regarding analyses within methods, we will conduct separate CFAs for each method (personality inventory, SJT and BDI). Regarding analyses across methods, we will conduct multitrait-multimethod CFAs on data from all three methods. The personality traits (Big Five traits plus Honesty-Humility) will be specified as latent trait factors and the three methods (personality inventory, SJT, and BDI) will be specified as latent method factors. Thereby, we will examine to which extent the different methods (personality inventory, SJT, and BDI) measure the same constructs.

In order to test the assumption that BDIs and SJTs both explain incremental variance in performance criteria over and above personality inventories, we will conduct construct-driven comparisons [see for example Lievens and Sackett (2006)] of personality measures predicting each criterion. To this end, we will conduct hierarchical regression analyses and relative weights analyses (Johnson, 2000) using the *relaimpo* package for R (Grömping, 2006). More precisely, we will conduct separate analyses for each performance criteria with the predictor constructs relevant for the specific performance criteria. As predictors, the respective personality constructs measured with different methods (i.e., personality inventory, SJT, and BDI) will be added to the model. Relative weights analyses will be used to test the hypothesis that personality traits assessed with the BDI are the strongest predictors of performance criteria (as compared to personality traits assessed with SJTs and personality inventories). Finally, we will test all hypotheses simultaneously in a path model using the *lavaan* package for R (Rosseel, 2012). This allows us to test hypotheses while accounting for the interdependencies among criterion constructs. The first author has already programmed the R script, which will be used to analyze data.

## Discussion

The aim of this study is to identify suitable approaches to personality assessment in the context of personnel selection for predicting a wide range of performance criteria. Personality has faced an up and down history in personnel selection, resulting in the conclusion that “personality constructs may have value for employee selection, but future research should focus on finding alternatives to self-report personality measures” (Morgeson et al., 2007, p. 683). Critics of the use of personality assessment for selection purposes further point to their low validities when predicting job performance (Morgeson et al., 2007). The proposed study is among the first to address this issue by systematically comparing different approaches to measure personality (personality inventory, SJT, BDI) to predict both task- and non-task performance dimensions. Specifically, we aim to enhance the criterion-related validity of personality constructs with two approaches. First, we develop measures with favorable features compared to personality inventories. We will vary different method characteristics, namely contextualization, source of information, and response-format. This modular approach was suggested in an earlier study because it allows for the systematic examination of the influence of measurement methods on criterion-related validity (Lievens and Sackett, 2017). Second, we shift the focus to non-task performance, thereby aiming to enhance the conceptual fit between personality predictors and performance criteria. Thus, this study aims to provide important insights on how to optimize the use of personality measures in the context of selection research and practice.

### Anticipated Results

We have three expectations regarding the results of this study. First, we expect different sets of personality constructs to predict task performance and especially different non-task performance criteria (i.e., adaptive performance, OCB, and CWB). Second, we expect that complementary measures of personality (i.e., SJTs and BDIs) will explain a significant proportion of performance criteria beyond personality inventories. Third, we expect BDIs to be superior to all other measurement methods in predicting all performance criteria. Specifically, we expect that personality constructs assessed with methods with a higher contextualization, which rely on self- and other-ratings and use an open-ended response format will be the strongest predictors of corresponding performance criteria. This implies that measuring personality with a BDI should lead to the strongest prediction, followed by SJTs and contextualized personality inventories.

Nevertheless, findings that are not in line with our assumptions could also generate valuable knowledge for research and practice. A different possible outcome of this study could be that SJTs and BDIs do not explain variance beyond personality inventories, or that the magnitude of difference in explained variance might be very small. If so, this could indicate that the respective method characteristics of SJTs and BDIs are not decisive for validity and selection research and practice would be advised to continue the use of personality self-report inventories (if assessing personality at all). Another different outcome could be that the variance explained by a measurement method depends on the traits that are measured (e.g., Extraversion might be better assessed with BDIs than with SJTs or personality inventories). This would imply that practitioners should base their choice of method based on the traits they aim to measure.

In each case, we hope that the findings of this study will encourage future research to examine alternative methods to measure personality in the context of personnel selection. If we find support for the assumption that specific method characteristics (e.g., open-ended vs. closed-ended response formats) affect the criterion-related validity of personality measures, future studies should further examine the mechanisms explaining *why* these method characteristics are particularly relevant. For example, the examined method characteristics could lead to differences in faking or applicant motivation, influencing the measurement of personality. Further, if SJTs and BDIs are suited to measure personality, an important next step will be to examine the fairness of different, but parallel designed measurement methods, for example by studying subgroup differences. This will help researchers investigate whether these measurement methods might have further favorable effects in personnel selection processes beyond their suitability to predict performance.

### Anticipated Limitations

A relevant limitation of this study is that participants will not be actual applicants. Thus, it might be that effects are not generalizable to a real selection setting (Culbertson et al., 2017; Powell et al., 2018). For example, participants in this study might feel less nervous compared to a real selection setting, because they are not applying for a real job. Further, they might behave less competitively in group-exercises, because they do not perceive other participants as their rivals. Yet, we chose this setting because it will allow us to compare a parallel personality inventory, SJT, and BDI all processed by each participant, with conditions close to a real selection setting. The setting further permits us to keep circumstances constant (e.g., interview rooms, schedule over the day of selection training, training of assessors and interviewers), thereby reducing error variance inherent to real selection settings. By creating an atmosphere close to reality (e.g., by asking both participants and assessors to wear professional clothes, by awarding a prize for the best participant) we will minimize the difference to a real selection process as much as possible. Yet, this limitation leads to a cautious estimation of criterion validity.

Even though we compare a number of important method characteristics, the comparisons in this study are not exhaustive. For example, we will compare open-ended and close-ended response formats (consent scales and single choice scales), but not other formats, such as forced-choice response formats, which are also used in personality testing (Zuckerman, 1971; SHL, 1993) and can positively affect validity (Bartram, 2007). Future studies using systematic comparisons of personality methods should consider further method characteristics, such as forced-choice formats.

### Practical Implications

Depending on the results, this study will inform practitioners about which set of personality traits they can use for the prediction of specific performance outcomes (e.g., adaptive performance). This would help them to design selection procedures purposefully in order to collect the information that is most helpful to predict the outcome of interest.

Further, this study will provide insights on which measurement method is most useful for assessing personality and predicting related outcomes in the context of personnel selection. These insights could help to better exploit the potential of personality in applied contexts. Specifically, the systematic comparison of three different personality measures (with varying method characteristics) that are designed in parallel to assess the same traits will provide detailed guidance on how to develop more valid personality measures in the future.

## Data Availability Statement

The original contributions presented in the study are included in the article, further inquiries can be directed to the corresponding author.

## Ethics Statement

The studies involving human participants were reviewed and approved by Ethics Committee of the Faculty of Arts and Social Sciences, University of Zurich. The participants provided their written informed consent to participate in this study.

## Author Contributions

All authors have shaped the research idea and study protocol. MK and PI developed the initial ideas. VS, AH, and MK planned the study in detail. VS wrote the study protocol. AH, PI, and MK provided substantial feedback in writing the study protocol.

## Conflict of Interest

The authors declare that the research was conducted in the absence of any commercial or financial relationships that could be construed as a potential conflict of interest.

## Footnotes

^1^We acknowledge that a relevant stream in the body of literature on adaptive performance examines employee performance before and directly after a task change and distinguishes transition adaptation and reacquisition adaptation (Lang and Bliese, 2009; Jundt et al., 2015; Niessen and Lang, 2020). Given that we aim to predict more generic adaptive behavior across different jobs with limited control over the nature of their task changes, the current study focuses on reactive and proactive forms of adaptive behavior.
